# DNA Content in Extracellular Vesicles Isolated from Porcine Coronary Venous Blood Directly after Myocardial Ischemic Preconditioning

**DOI:** 10.1371/journal.pone.0159105

**Published:** 2016-07-19

**Authors:** Kristina Svennerholm, Pouria Rodsand, Urban Hellman, Anders Waldenström, Marie Lundholm, Dag Ahrén, Björn Biber, Gunnar Ronquist, Michael Haney

**Affiliations:** 1 Anesthesiology and Intensive Care Medicine, Institute of Clinical Science, Sahlgrenska Academy, University of Gothenburg, Gothenburg, Sweden; 2 Anesthesiology and Intensive Care Medicine, Institute of Surgical and Perioperative Science, Umeå University, Umeå, Sweden; 3 Cardiology, Institute of Heart Centre and Department of Public Health and Clinical Medicine, Medicine, Umeå University, Umeå, Sweden; 4 Pathology, Institute of Medical Biosciences, Umeå University, Umeå, Sweden; 5 National Bioinformatics Infrastructure Sweden (NBIS), Institute of Biology, Lund University, Lund, Sweden; 6 Clinical Chemistry, Institutet of Medical Sciences, Uppsala University, Uppsala, Sweden; University of Louisville, UNITED STATES

## Abstract

**Background:**

Extracellular vesicles (EV) are nano-sized membranous structures released from most cells. They have the capacity to carry bioactive molecules and gene expression signals between cells, thus mediating intercellular communication. It is believed that EV confer protection after ischemic preconditioning (IPC). We hypothesize that myocardial ischemic preconditioning will lead to rapid alteration of EV DNA content in EV collected from coronary venous effluent.

**Materials and Methods:**

In a porcine myocardial ischemic preconditioning model, EV were isolated from coronary venous blood before and after IPC by differential centrifugation steps culminating in preparative ultracentrifugation combined with density gradient ultracentrifugation. The EV preparation was validated, the DNA was extracted and further characterized by DNA sequencing followed by bioinformatics analysis.

**Results:**

Porcine genomic DNA fragments representing each chromosome, including mitochondrial DNA sequences, were detected in EV isolated before and after IPC. There was no difference detected in the number of sequenced gene fragments (reads) or in the genomic coverage of the sequenced DNA fragments in EV isolated before and after IPC. Gene ontology analysis showed an enrichment of genes coding for ion channels, enzymes and proteins for basal metabolism and vesicle biogenesis and specific cardiac proteins.

**Conclusions:**

This study demonstrates that porcine EV isolated from coronary venous blood plasma contain fragments of DNA from the entire genome, including the mitochondria. In this model we did not find specific qualitative or quantitative changes of the DNA content in EV collected immediately after an *in vivo* myocardial IPC provocation. This does not rule out the possibility that EV DNA content changes in response to myocardial IPC which could occur in a later time frame.

## Introduction

Extracellular vesicles (EV) are nano-sized membranous structures released from most cells. The EV have emerged as a new mechanism for cell-to-cell communication, by serving as vehicles for transfer of bioactive molecules and RNA sequences between cells in an organism [1–3]. The term EV includes different membrane enclosed vesicles, exosomes, microvesicles and apoptotic bodies. While these different subgroups are different in size, there have until recently not been specific markers that distinguish subsets of EV from each other [4]. Categorization of EV has been based on successive centrifugations, flotation into iodixanol gradients (displaying different buoyant densities), or immune-isolation followed by comprehensive proteomic analysis of isolated subgroups [5].

EV have been shown to carry DNA fragments [6–8]. The first report of DNA occurrence in EV was in 1990, in human epithelial prostate cells [6]. Subsequent characterization of the prostasomal DNA showed fragments from the entire genome [9–11]. Other groups identified mitochondrial DNA as well as genomic DNA in EV released by glioblastoma cells in cultures and tumor cells *in vivo* [12, 13]. More recently, EV from a broad panel of cancer cell lines and from plasma of patients with pancreatic cancer have been shown to contain DNA fragments which represent the entire genome, including the mutation of the cellular tumor source [8, 14].

There are several reports suggesting that cells receive and internalize EV, and that there is a functional impact of the transferred nucleic acid content [1, 7, 15]. The environment of the parental cell is apparently influential on the messenger RNA (mRNA) content within EV that the cell elaborates [15–17]. The biological role of DNA transfer between cells, as well as the dynamics in EV DNA content, is not yet well understood. However, it was demonstrated that EV from murine cardiomyocytes and EV from human plasma contain thousands of distinct genomic DNA fragments that were transferable into the nucleus of recipient cells [7, 18]. Moreover, a physiological significance was identified when transfer of human EV DNA resulted in increased transcription and production of mRNA and proteins, thus demonstratig that EV genetic contents can influence the function of recipient cells *in vitro* and *in vivo* [19]. A biological effect of EV DNA has been reported when rat epithelial cells, transformed by human oncogenes, generated EV that contained DNA fragments covering the entire rat genome, including the human oncogene. When non-transformed cells internalized these EV, it led to cell proliferation [20].

EV have recently been suggested as endogenous mediators of cardioprotection, both concerning myocardial ischemic preconditioning (IPC) as well as local remote IPC [21–23]. Myocardial IPC is a phenomenon of endogenous cardioprotection through adaptive tolerance of myocardial ischemia. The treatment includes short and repeated periods of myocardial ischemia, followed by brief phases of reperfusion and leads to an increased cellular ischemia tolerance by reduction of infarct size during a subsequent prolonged myocardial ischemic period [24]. Studies have shown that EV might mediate protection in ischemic or infarcted myocardium by attenuating infarct size and/or improving cardiac function *in vivo* [25–27]. Specifically, micro RNA (miRNA) content of EV has been explored in these contexts [21, 26, 27]. In a previous *in vivo* myocardial IPC study, rapid changes in coronary venous EV mRNA content were observed [28].

In the present study we hypothesized that DNA can be identified in porcine plasma-derived EV as well as that myocardial IPC leads to changes in EV DNA content. We aimed to test this using an *in vivo* porcine model of myocardial IPC with EV collection from regional cardiac venous effluent before and directly after the intervention.

## Materials and Methods

The study was approved by the Regional Animal Research Ethics Committee in Umeå, Sweden (dnr: A182-12), and conducted in adherence with Guide for the Care and Use of Laboratory Animals (National Academy of Science, USA 1996) and the EU Directive 2010/63/EU for animal experiments (http://ec.europa.eu/environment/chemicals/lab_animals/legislation_en.htm).

Swedish land-race pigs, which were raised and supplied for research purposes by a local school of agriculture, were premedicated with a mixture of ketamine 10 mg/kg^-1^ (Ketalar, Pfizer, Morris Plains, New Jersey, USA) and xylazine 2 mg/kg^-1^ (Rompum vet, Bayer AB, Lyngby, Denmark) intramuscularly, followed by induction of anesthesia with intravenous (iv) pentobarbital 10 mg/kg^-1^ (Pentobarbital natrium, Apoteksbolaget, Stockholm, Sweden). A continuous iv infusion of pentobarbital 5 mg/kg^-1^/h^-1^, fentanyl 20 ug/kg^-1^/h^-1^ (Fentanyl, Braun, Melsungen, Germany) and midazolam 0,3 mg/kg^-1^/h^-1^ (Dormicum, Roche, Basel, Switzerland) (without use of muscle relaxants) was used for maintenance of anesthesia. Ringer’s acetate was infused at 15 ml/kg^-1^/h^-1^ throughout the protocol and core body temperature was maintained at 38–39°C. Animals were tracheostomized and mechanically ventilated (Evita 4, Dräger, Kiel, Germany) (volume-controlled mode) to normoxia and normocapnia. Mean arterial pressure, ECG and central venous pressure were observed and recorded continuously during the experiment. The animals were euthanized by a bolus of pentobarbital and potassium chloride at the end of the experiment.

### Preparation

Neck artery and veins were cannulated following surgical dissection. A triple-lumen central vein catheter (Arrow-Howe Multi-Lumen Central Venous Catheter, Vingmed, Järfälla, Sweden) was positioned for CVP measurements and infusion of drugs and fluids. For invasive blood pressure an arterial catheter was placed and for sampling blood from coronary veins draining the myocardial ischemic area, a coronary sinus catheter (CCS-7 U-90A; Webster Labs, Altadena, California, USA) was positioned by using flouroscopic guidance. Through midline sternotomy followed by pericardiotomy, a patched snare was placed around the middle portion of the left anterior descending artery (LAD) for intermittent occlusion. Myocardial ischemia and accurate reperfusion sampling was confirmed by more than doubling of coronary lactate production, measured in coronary venous blood. Animals were allowed to rest for one hour after surgical preparation, before baseline blood samples were collected.

The protocol of myocardial IPC has been described previously where the treatment effect is generated by temporary snare-occlusion for 10 min followed by reperfusion during 20 min, repeated in four cycles [29]. Coronary venous sampling after IPC, were collected during a period of 20 min, starting 20 min after last LAD snare release. Blood samples were immediately centrifuged (10 min, at 4°C and 1800 x g) and resulting plasma was further mixed with an equal amount of phosphate-buffered saline (PBS). Samples were stored in –80°C until thawed in room temperature prior to EV isolation.

### Extracellular vesicle isolation

**Fig 1:**
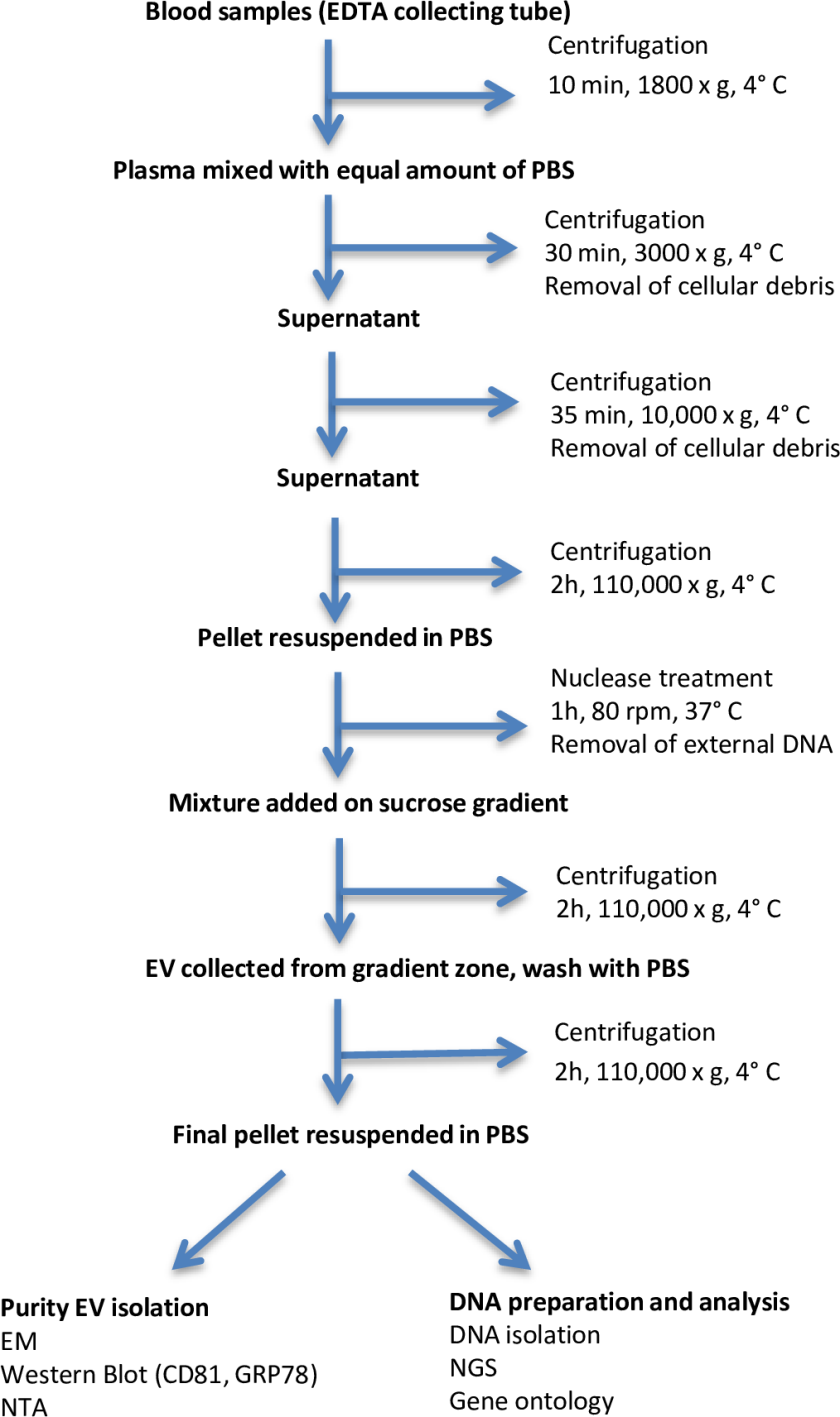
Work-flow diagram of EV isolation from porcine blood, followed by evaluation of EV purity and DNA isolation and analysis.

EV were isolated from the buffer-diluted plasma samples by differential centrifugation steps followed by preparative ultracentrifugation. Samples were centrifuged for 30 min at 4°C and 3,000 x g, and then at 10,000 x g for 35 min at 4°C to remove possible cellular debris. In order to gather EV in a pellet, the supernatants were ultracentrifuged for 2 h at 110,000 x g and 4°C. The EV pellets were re-suspended in 1.5 mL phosphate buffered saline (PBS) followed by nuclease treatment (Benzonase Nuclease Ultrapure, ≥250 Units/μl, Sigma-Aldrich, Brøndby, Denmark), for 1 h at 37°C and 80 rpm, for removal of possible external RNA and DNA. For removal of contaminating material, EV mixtures were immediately added on top of a sucrose gradient (prepared by equal amounts of 20% and 40% sucrose solution) and then ultracentrifuged. EV were collected from the gradient zone (density range 1.13–1.19 g/mL) and then washed with PBS before last ultracentrifugation at 110,000 x g for 2 h and 4°C. All steps of ultracentrifugations were ran at 110,000 x g for 2 h and 4°C using a L-90 Beckman centrifuge and the SW-41 rotor (Beckman Instruments, Inc., Fullerton, CA). The final EV pellets were resuspended in PBS before storage at– 80°C (Fig 1).

### Evaluation of extracellular vesicle preparation

Electron microscopy (EM), nanoparticle tracking analysis (NTA) and western blot (described below) were conducted in order to confirm presence of EV as well as absence of contaminants such as larger vesicles and cells. EM of isolated EV was performed at the EM unit Emil, Clinical research center, Stockholm, Sweden. The NTA (NanoSight N300, Malvern Instruments Ltd. Malvern, United Kingdom) was performed on final EV preparation samples diluted in PBS (1:500–1:1000). Western blot analysis was performed on lysed EV and cell suspension of crushed porcine myocardial tissue. CD81 was used as EV marker and GRP78 to detect potential contamination of cells and apoptotic bodies. It is suggested to investigate more proteins of an EV preparation, but due to few available porcine antibodies, we were restricted to quantify only these two.

### Western blot

Protein concentration was determined using BCA Protein assay (Pierce) and samples of 5–10 μg protein were separated by 10% and 5–14% Mini-PROTEAN TGX gels (Bio-Rad, USA). The separated protein fractions in the gels were then transferred to PVDF membranes (Midi format 0.2 μm, Bio-Rad) using Trans-Blot Turbo (Transfer System, Bio-Rad). The membranes were blocked by Odyssey Blocking Buffer (LI-COR) for 1 h at 21°C, washed with PBS Tween (PBST) and subsequently incubated with anti-CD81 antibody (Santa Cruz Biotechnology, Texas, USA, diluted 1:200 in 1% PBST) and anti GRP-78 antibody (Abcam, England, diluted 1:1000 in 1% PBST) at 4°C overnight. The membranes were washed with PBST before they were incubated with secondary Goat anti-rabbit antibody (IRDye, 800 CW, LI-COR, diluted 1:10 000 in 1% PBST) for 1 h at 21°C. After washing with PBST the proteins were visualized using LI-COR ODYSSEY GLx.

### Extracellular vesicle DNA extraction

To isolate DNA from the EV preparation, the The Qiagen Allprep DNA/RNA Mini kit (Qiagen, Alameda, CA, USA) was used according to the manufacturer’s instructions. DNA was stored in -80° C.

### DNA sequencing

The EV DNA sequencing was performed by National Genomics Infrastructure, NGI, Uppsala, Sweden. An IonProton instrument was used with a standard/whole genome Ion DNA library and a Proton I, 10 Gb chip and a reading length of 200 base pairs of each original EV DNA sequence. The number of times each 200 base pairs unique DNA sequence is detected, is identified as the number of reads for that specific sequence. The EV DNA from all pigs before IPC was pooled, and the same was done for the EV DNA material after IPC, before the DNA sequencing in order to retrieve enough DNA quantity.

### Bioinformatics

The bioinformatical analysis of the sequence data was performed in consultation with the Bioinformatics Infrastructure for Life Sciences (Uppsala, Sweden). The computing was performed at Uppnex [30]. Mapping of the FASTQ files against the Sus scrofa genome version 10.2 was performed using gsMapper version 2.9. with default settings and bam output chosen. The bam files were converted to bed files and intersected with gene annotations (GFF3) from Sus scrofa genome at the NCBI website. The number of reads of each gene detected in the sequenced regions was calculated using intersectBed from the Bedtools programs version 2.23. The output results were subsequently parsed using an in house script, and the most abundant genes were investigated further.

### Gene ontology

Gene ontology analysis was performed on the 3000 porcine genes with the most reads in EV isolated before IPC. This was compared to gene ontology analysis performed on the 3000 porcine genes with the most reads in EV isolated after IPC. The number of reads corresponds to the number of times a certain gene has been sequenced during the analysis. Gene ontology analysis was performed using the functional annotation clustering tool in the web-accessible program DAVID Bioinformatics version 6.7 (http://david.abcc.ncifcrf.gov/home.jsp). All options in the analysis were set on default. Clusters having enrichment score >1.3 were regarded as significant [31].

## Results

### Purity of extracellular vesicle preparation

**Fig 2:**
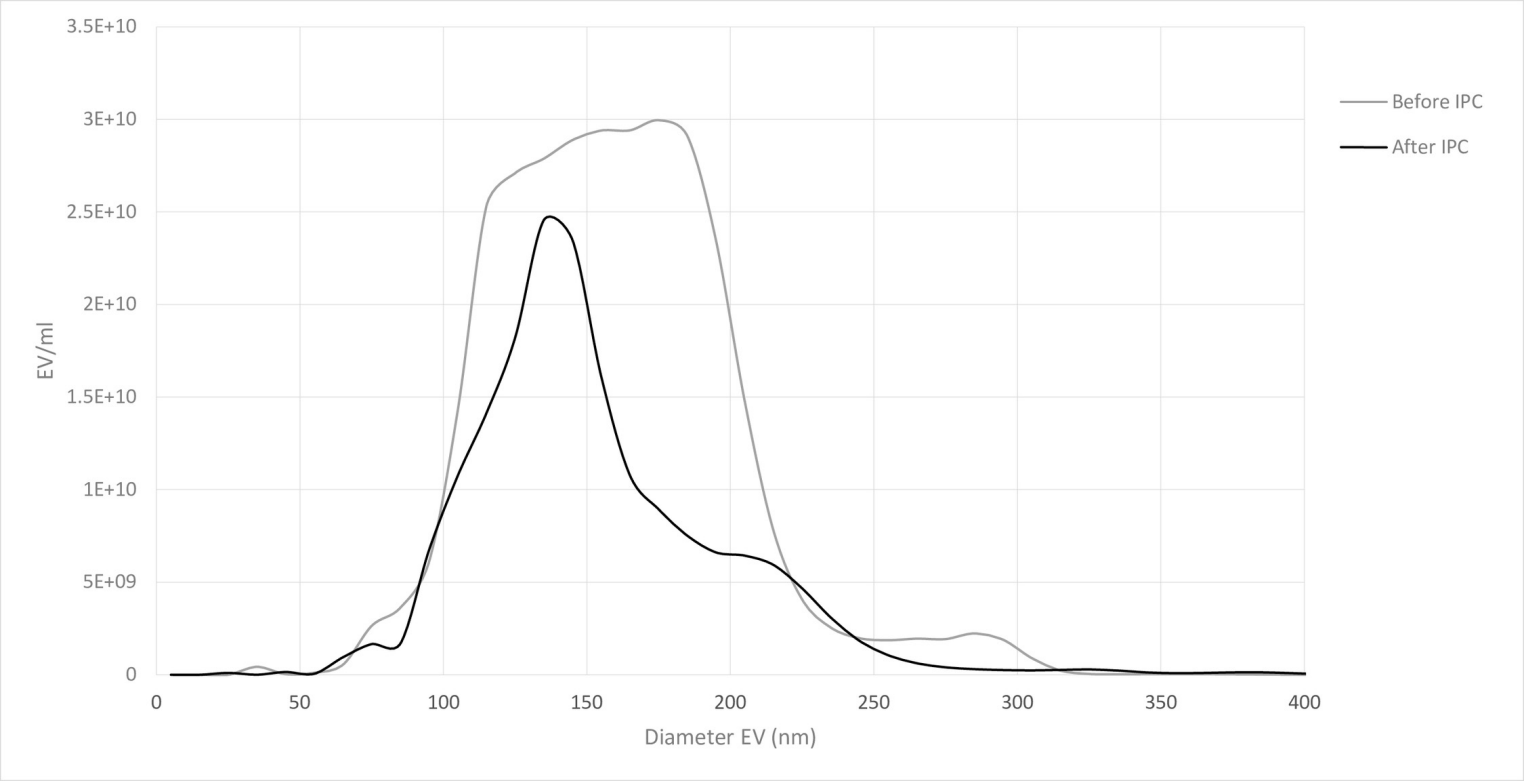
Nanotracking analysis. EV size distribubution before and after IPC.

Five animals (all approximately 40 kg) completed the IPC protocol, with coronary venous blood samples collected before IPC and after the 4^th^ IPC cycle, 20 min after coronary snare release. EV were isolated from the porcine plasma using the standard sequential ultracentrifugation method. To confirm and validate the presence of EV, the EV preparations were evaluated by western blot, Nanoparticle tracking analysis (NTA) and electron microscopy (EM). CD81, a known tetraspanin protein enriched in EV, was detected in EV samples but not in the porcine cell suspension, which indicates that EV preparations were correctly purified. GRP78, an endoplasmic reticulum protein, was detected in cell suspension but not in the EV preparation, previously published [28]. This indicates absence of contaminating apoptotic bodies. NTA showed EV ranging from 30 to 350 nm, the major part within the range 75 and 200 nm (Fig 2). EM analysis of the EV fractions demonstrated a typical ultrastructure of an EV of similar size as detected by NTA and absence of cell debris as previously presented in the method section of an earlier pulication [28].

### Gene fragments

Porcine genomic DNA from EV was analysed in the samples from before and after IPC. The DNA sequences in both samples represented all chromosomes of the porcine genome, including porcine mitochondrial DNA. Data and information are available throw NCBIs sequence read archive (SRA) and are accessible throw SRA, BioProject ID: PRJNA325580

### Chromosome location and gene fragment quantification

**Fig 3:**
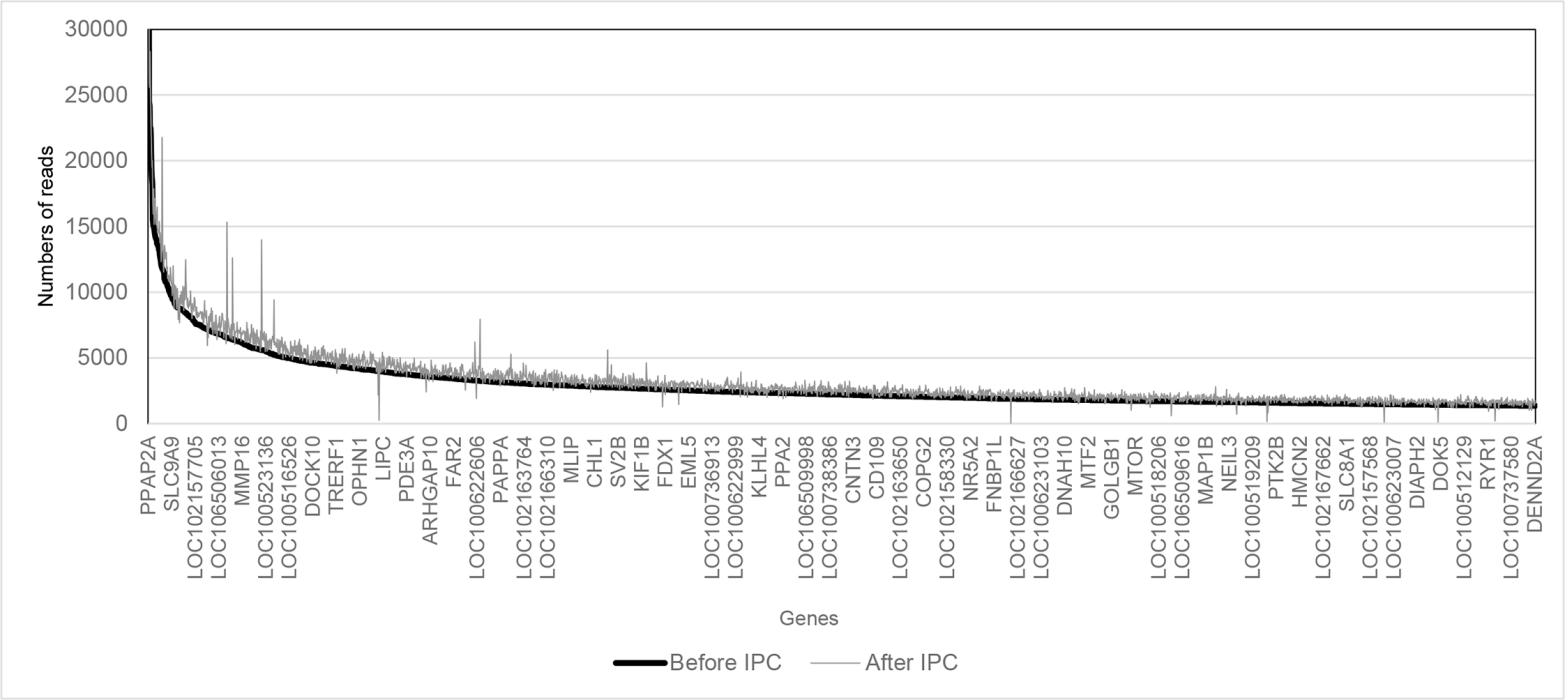
Gene fragment quantification. The Y-axis shows the number of sequenced gene fragments (reads) isolated in EV before (black) and after IPC (grey). The x-axis shows the 3000 genes (not all named) with most reads in decreasing number of read frequency before IPC. The genes detected after IPC are presented in the same order as the genes before IPC, in order to illustrate possible differences for numbers of reads. Due to the high number of genes, the x-axis shows only a few of these (S2 Fig for the complete data). The lines are closely located and do not show any significant difference between before and after IPC.

The genome coverage analysis for all chromosomes did not detect any significant difference in the location of the alignments between EV DNA before and after IPC (S1 Fig). There was an average increase of approximately 400 reads per gene after IPC. The systematically continuous increase in reads for each gene is most likely a result of technical or analytical rather than a biological cause. Otherwise, there was no significant difference in the number of reads in EV isolated before IPC compared to EV isolated after (Fig 3).

#### Gene ontology

Although gene ontology was performed on the 3000 porcine genes with the most reads, DAVID could only identify the function of 278 of those 3000 genes (S2 Fig). The analysis showed that the genes with most reads in EV before IPC were coding for the same clusters of proteins as DNA in EV after IPC. The analysis detected an enrichment of DNA coding for ion channels, enzymes and proteins involved in basal metabolism, vesicle biogenesis and specific cardiac proteins.

## Discussion

The initial finding of this study was that pig genomic DNA fragments were identified in coronary venous plasma-derived EV from an *in vivo* porcine model. Furthermore, porcine mitochondrial DNA was detected in these EV. The DNA sequences detected in EV representing all chromosomes of the porcine genome is a novel finding. These findings support the idea of a general presence of DNA fragments in EV.

The main finding is that the DNA content in coronary sinus plasma-derived EV did not change directly after the *in vivo* myocardial IPC provocation. The numbers of reads (for coding genes) were equal in EV isolated before and after IPC. Further, the genomic location on each chromosome was unchanged by the myocardial IPC treatment. Both the number of reads and the genomic location of the reads showed minor differences in EV isolated after IPC, although it is not possible to rule out technical reasons or normal biological variation as the cause for these small variances. These findings did not support the study hypothesis that early post-IPC EV genetic content would change. The origin of this specific hypothesis was in a previous study [28] were it was demonstrated that the mRNA contents in EV changed in early sampling with this myocardial IPC model. It is plausible that microRNA (miRNA) and messengerRNA (mRNA) alterations can be observed in EV at an earlier time compared to free DNA in EV after a cell stimulation or insult. The temporal relation of the appearance of different types of newly incorporated genetic material (miRNA, mRNA, DNA) in EV after a cell insult or injury has not yet been determined.

The gene ontology analysis (of the 3000 genes with most reads) showed an enrichment of genes in pathways and functional processes that did not differ between EV isolated before and after IPC. Among the significant enrichments, genes coding for ion channels, general proteins involved in basal metabolism and vesicle biogenesis were detected, but also, genes connected to the heart were found (S3 Fig). These cardiac gene sequences were the only organ specific fragments detected in the enrichment analysis. The finding is interesting and implies that the EV DNA is enriched of genes important for the specific parental cell, in this study the cardiomyocyte. However, this hypothesis has to be investigated in further studies to confirm this conclusion. The enrichment of cardiac gene sequences supports an interpretation that the methodology in this study was appropriate for isolation of EV originating primarily from the heart. Currently, there is no method to distinguish or separate EV of cardiac origin in plasma from EV originating from other cells or organs.

This study was designed to identify DNA fragments in porcine plasma-derived EV and possible changes in EV DNA content related to myocardial IPC, with the clinical implication that if such EV content related to protective effects of IPC could be identified, then this might open a new therapeutic alternative for cardioprotection. Recently, genomic DNA detected in EV obtained from human plasma was shown to be physiologically active and able to generate functional mRNA and proteins in recipient cells *in vitro* and *in vivo* [19]. Although the EV DNA content does not seem to be involved in the early protective effect of IPC, it is still possible that EV DNA content changes can appear later after cell ‘conditioning’, possibly many hours or days after a diffuse cell insult or IP-like treatment. One can speculate that EV DNA may have a biological function or role in an adaptive response by affecting phenotype of recipient cells. Further studies will be needed to assess functional implications of EV DNA in this context, where a protective effect might be demonstrated by EV DNA resulting from IPC.

The origin of DNA identified inside EV have so far not been explained. How nuclear DNA can find its way into small EV formed in the cytoplasm is difficult to imagine. Interestingly, the existence of cytoplasmatic membrane-associated DNA outside the nucleus has been verified, together with a special cytoplasmatic transcription system with RNA polymerase transcribing these DNA sequences [32]. Since the source of the EV membrane is the cell membrane it may be possible that the EV contain biological material from inside and close to the cell membrane and therefor also DNA. The question then is not why DNA is inside EV, it is why DNA is on the inside of the cell membrane. Perhaps this is a remnant from an ancient pre-nuclear cell replicating system.

There has been speculation about the biological role of horizontal DNA transfer between cells of an organism, since all cells of the organism are carrying an identical DNA composition. Some are argued that EV DNA is an artifact emerging from contaminants such as viruses, and others that the intercellular DNA transfer is not related to a biological function. Reports have recently demonstrated that genomic DNA in EV can be functional in recipient cells [19, 20]. A possible teleological explanation of the biological purpose of an intercellular DNA transfer might be that an increase in DNA content within a recipient cell also leads to an increase in gene transcription activity and ensuing protein production of that specific sequence. In our study, during EV isolation, the EV pellets were treated with nuclease to remove any possible DNA exterior to vesicles. The identified DNA fragments aligned specifically to the porcine genome, and this rules out the possibility of contamination. Therefore, we are confident about the authenticity of the described DNA present inside our EV.

The principle of DNA presence in EV seems to be consistent between species. Previous studies have demonstrated the existence of DNA in EV from humans and rodents [8, 12, 14, 20], but not in pigs. Our original observations on EV and DNA in pigs are of general interest since the pig is a relevant animal model sharing many physiological similarities with humans, and therefore, these findings can provide basis for future EV *in vivo* studies. Furthermore, knowledge of the function of all genes in the entire pig genome is essential for more detail studies. Testing of this type in the pig model can be advantageous since an injury or preconditioning intervention can be carefully controlled, and invasive and regionalized sampling (beyond just blood plasma sampling) is possible.

In summary, EV with their inclusion of DNA fragments, were clearly demonstrated in coronary venous samples from a porcine model undergoing myocardial IPC. The EV DNA content changes were not observed from sampling early after the ischemic provocation, even though there have been previous findings of new EV mRNA content in the same time frame and porcine myocardial IPC model. We could demonstrate a persistent DNA content in EV enriched with genes coding for specific heart proteins. We conclude that further studies are warranted to evaluate different signals within EV in IPC, as well as understanding the purpose of DNA transportation in EV.

## Supporting Information

S1 Fig Gene coverage analysis. (TIF)

S2 Fig Gene ontology on genes with the most reads. (ZIP)

S3 Fig Significant enrichments of gene ontology analysis. (TIFF)
