# Supplementary figures and images for: DNA Content in Extracellular Vesicles Isolated from Porcine Coronary Venous Blood Directly after Myocardial Ischemic Preconditioning
Source: PLoS One. 2016 Jul 19;11(7):e0159105. doi: 10.1371/journal.pone.0159105 (PMC4951030; doi:10.1371/journal.pone.0159105)

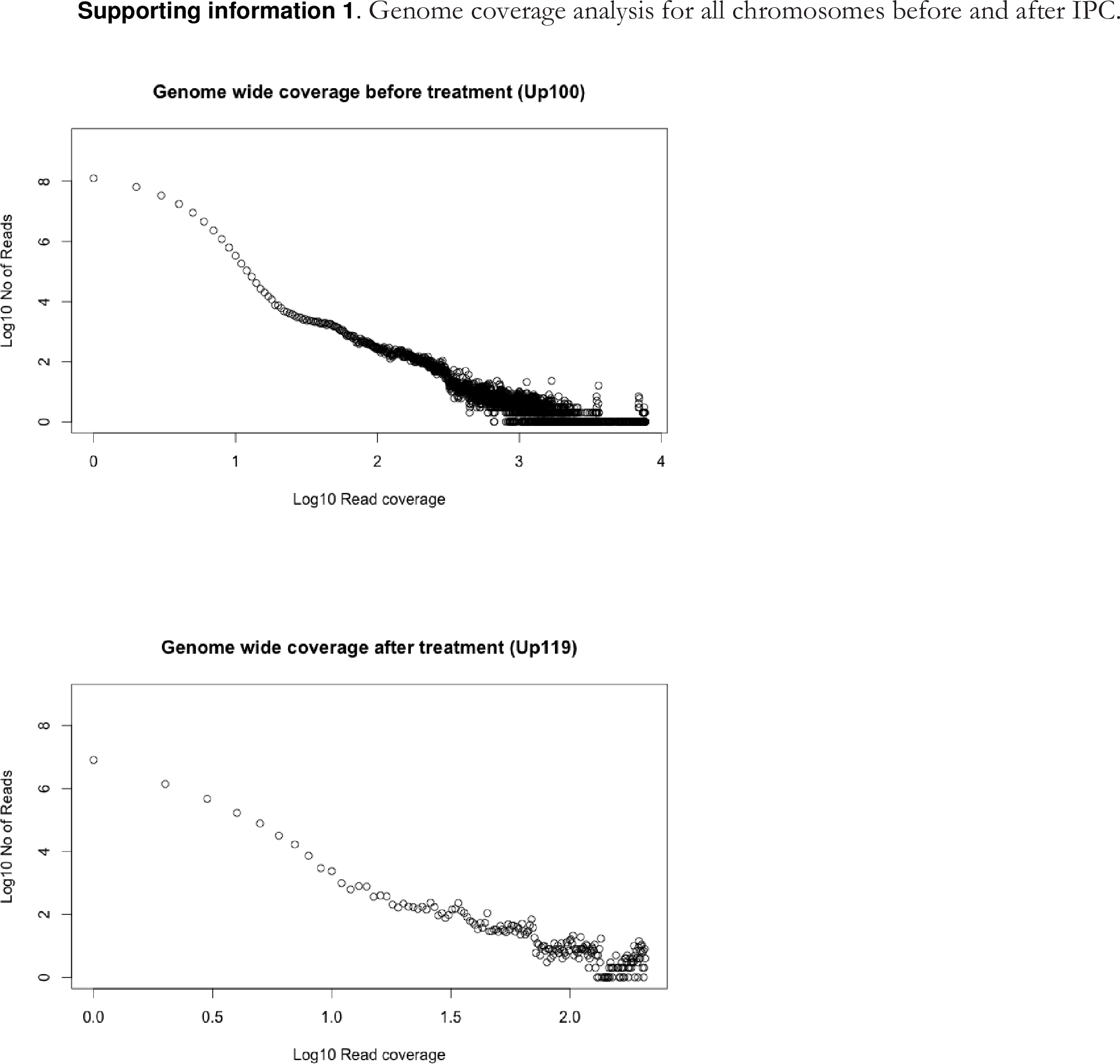

Supplement: S1 Fig — (TIF) [file pone.0159105.s001.tif]

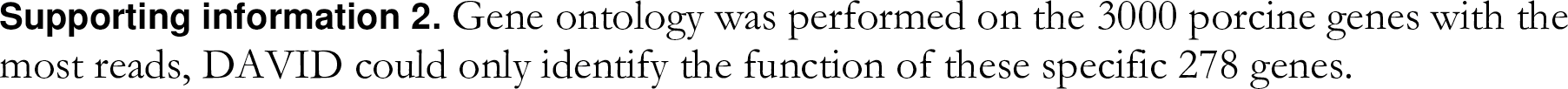

Supplement: S2 Fig — (ZIP) [file pone.0159105.s002.zip › S1 supporting information 2/S2A Fig.tif]

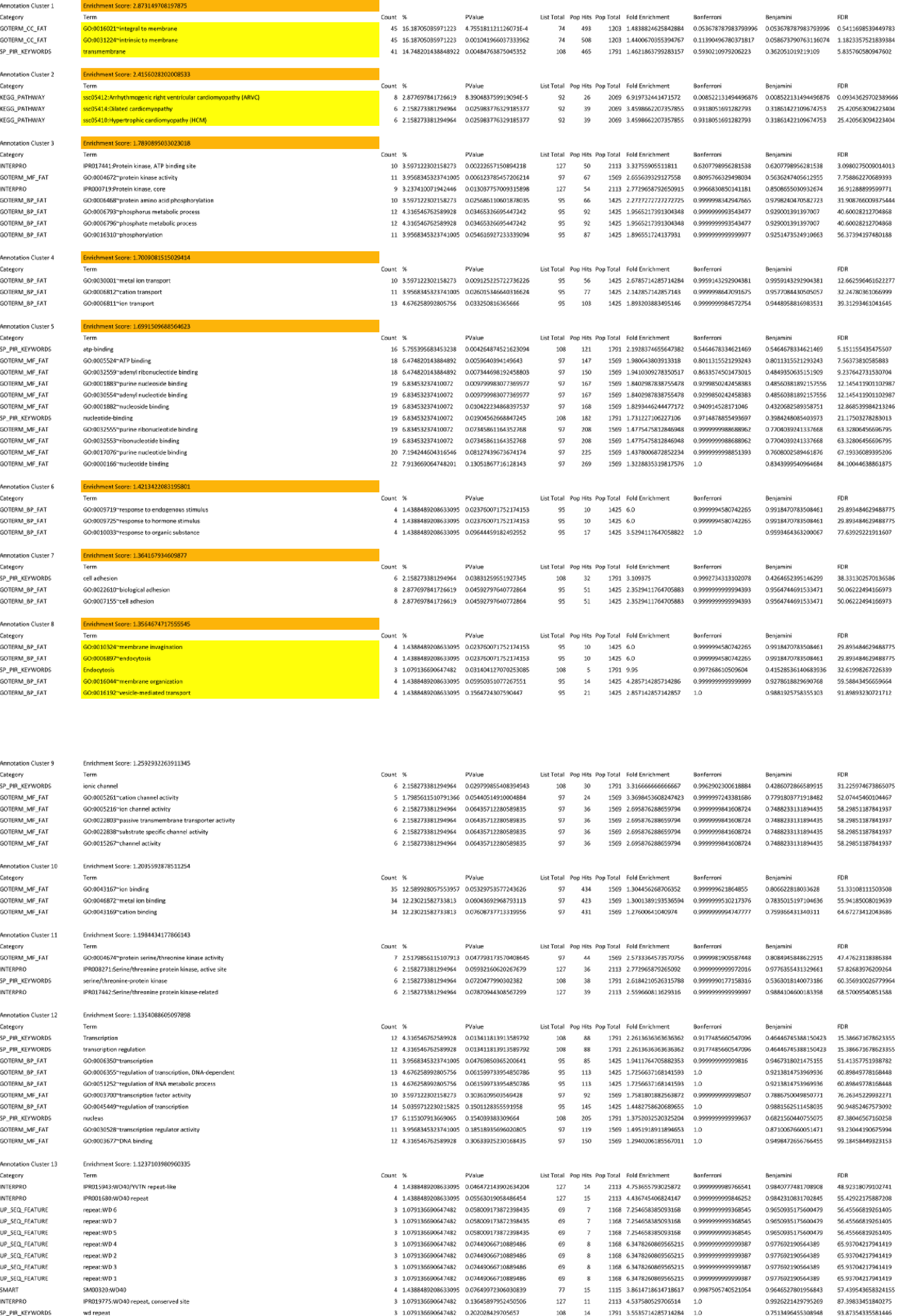

Supplement: S2 Fig — (ZIP) [file pone.0159105.s002.zip › S1 supporting information 2/S2B Fig.tif]

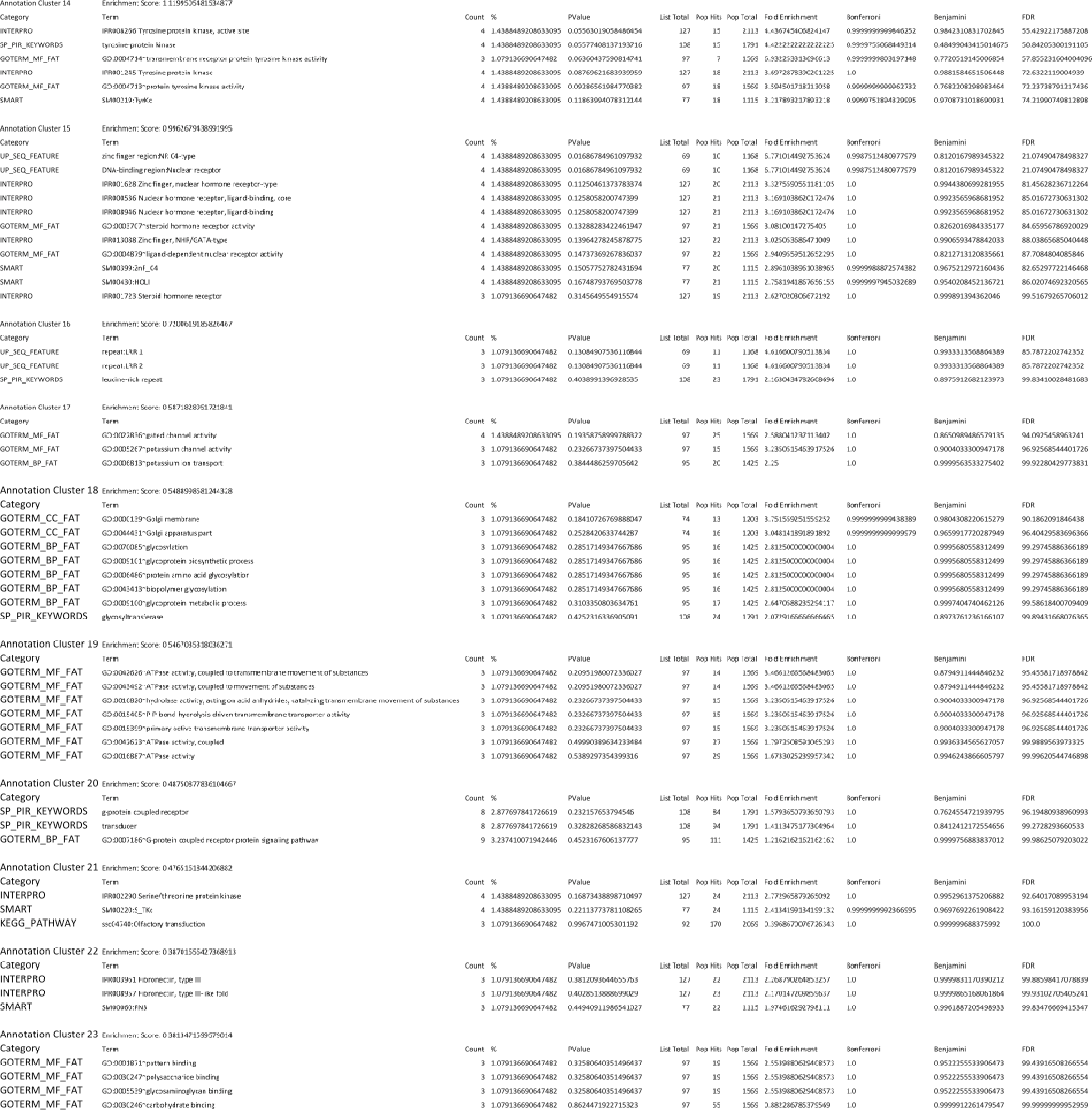

Supplement: S2 Fig — (ZIP) [file pone.0159105.s002.zip › S1 supporting information 2/S2C Fig.tif]

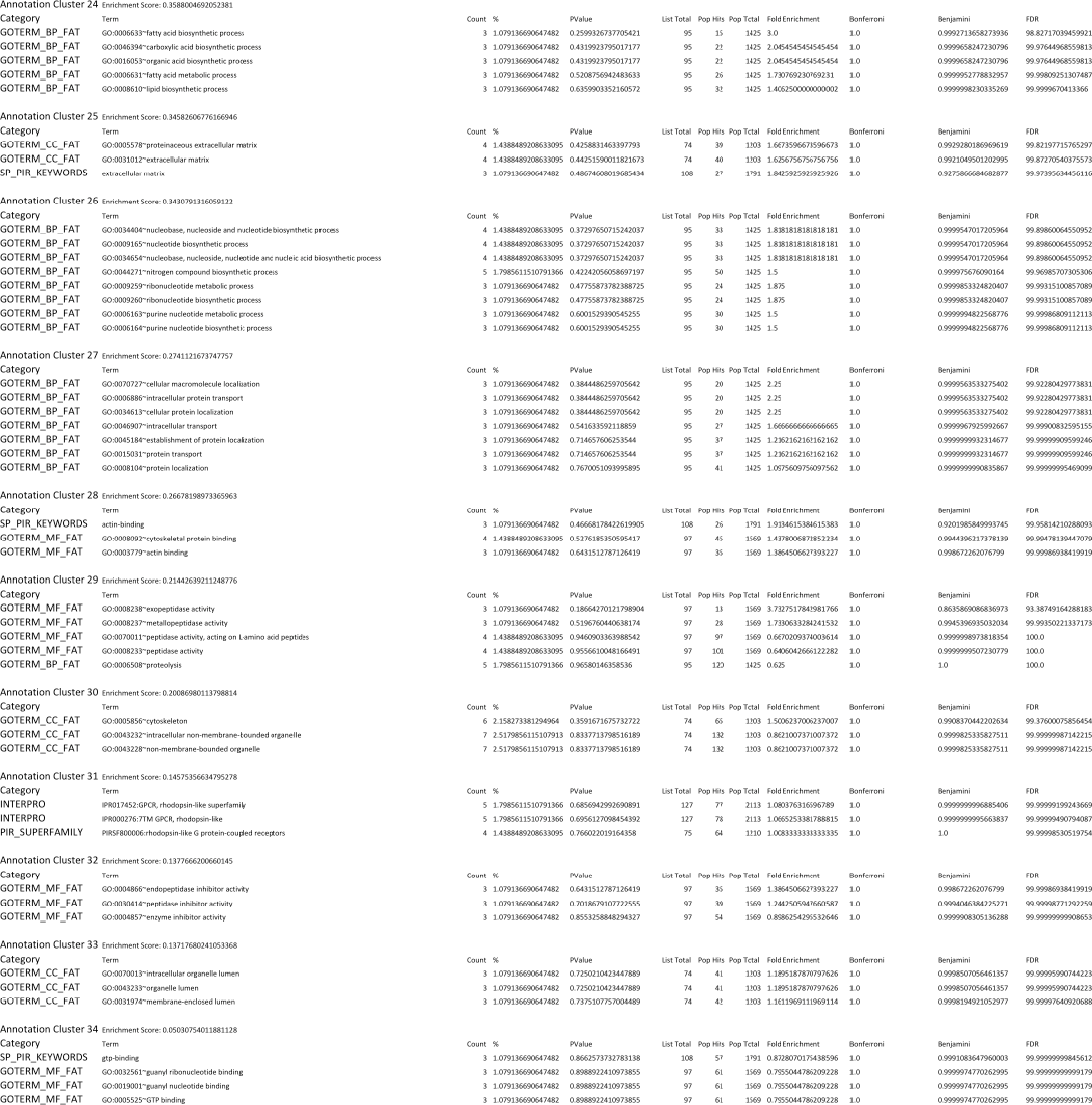

Supplement: S2 Fig — (ZIP) [file pone.0159105.s002.zip › S1 supporting information 2/S2D Fig.tif]
